# First person – Cloé Hadjadji and Molly Ohse

**DOI:** 10.1242/bio.062968

**Published:** 2026-10-29

## Abstract

First Person is a series of interviews with the first authors of a selection of papers published in Biology Open, helping researchers promote themselves alongside their papers. Cloé Hadjadji and Molly Ohse are co-first authors on ‘ High-throughput mitochondrial aerobic metabolism measurement: assessing a plate-reader approach using avian blood cells’, published in BiO. Cloé is a PhD student in the lab of Dr Pierre Bize at Swiss Ornithological Institute, Sempach, Switzerland and part of the Department of Ecology and Evolution, University of Lausanne, Switzerland, investigating the interface between ecology and physiology: how environmental stressors affect individual performance and, ultimately, populations. Molly is also a PhD student in the lab of Dr Pierre Bize at Swiss Ornithological Institute, and part of the Department of Ecology and Evolution, University of Lausanne, investigating how mercury and other metal contaminants affect the physiology and life history of wild birds.

**Figure:**
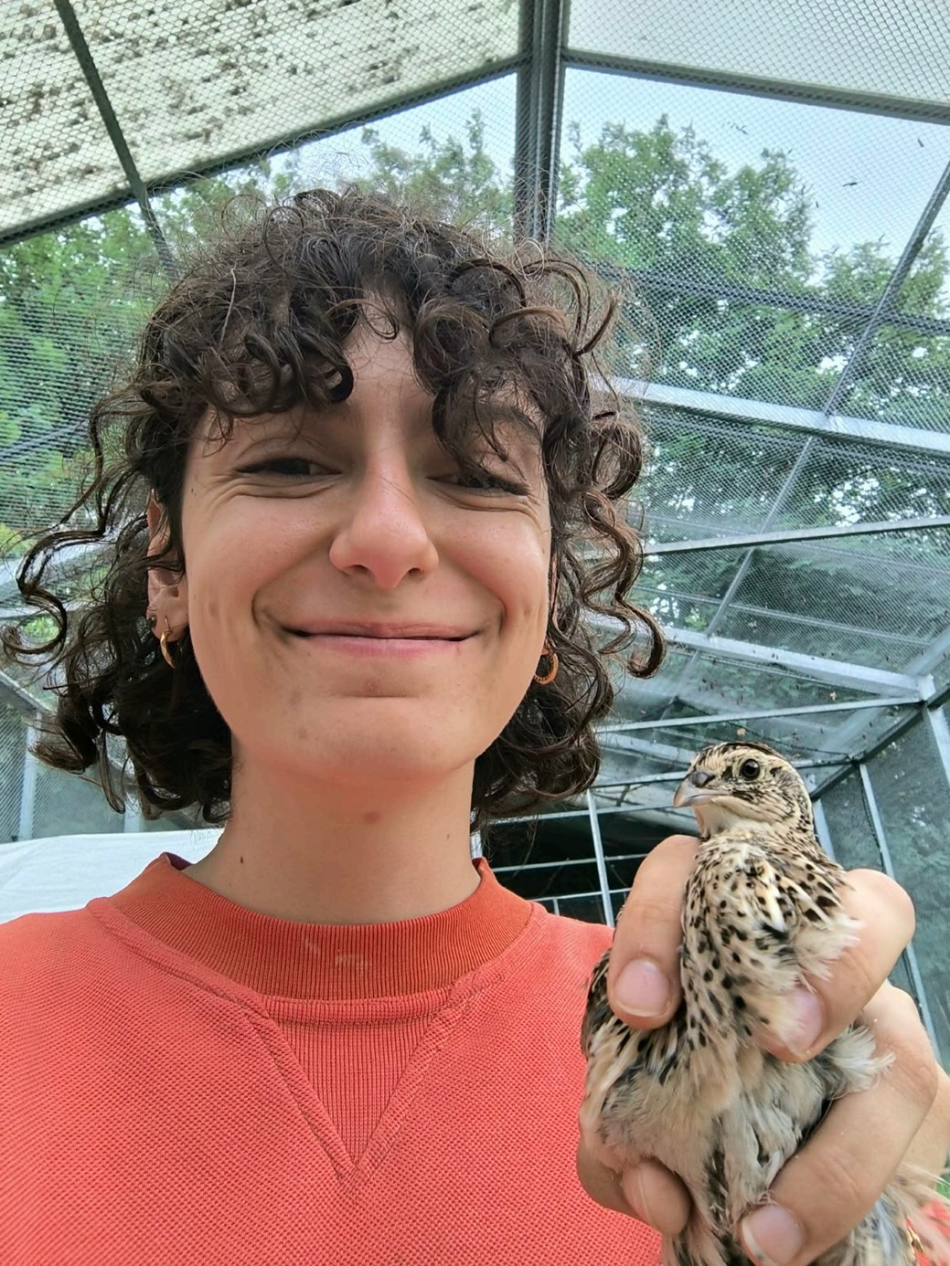
Cloé Hadjadji

**Describe your scientific journey and your current research focus**

**C.H.:** I did a Bachelor's in Life Sciences and then a Master's in Conservation Biology at the Université Marie et Louis Pasteur in France. Internships during those years shaped my research interests: first monitoring trace metal contamination and its effects in birds and mammals, then studying how urbanisation affects bird physiology. This led me to a PhD at the Swiss Ornithological Institute and the University of Lausanne, where I study selection on and heritability of mitochondrial traits in the alpine swift, and how these traits relate to other aspects of physiology, and whether they predict fitness.

**M.O.:** I studied conservation biology at the University of Aberdeen in Scotland, where my Bachelor's honours project first sparked my interest in how contaminants affect wild birds. I stayed in Aberdeen for my Master's, which gave me my first chance to study this in a wild population. I then built on that project for my PhD at the Swiss Ornithological Institute and the University of Lausanne in Switzerland. Working with a long-term, individual-based European dipper population in the Chartreuse Mountains of France, I investigate how mercury and other metals affect mitochondrial function, oxidative stress, and stress hormones, and whether these effects carry through to growth, reproduction, and survival.

**Who or what inspired you to become a scientist?**

**C.H.:** For as long as I can remember, I have been fascinated by nature and by living organisms of every kind. As a kid, I spent my time either outside exploring or inside reading books and watching wildlife documentaries. For a while I wanted to become a biology teacher or a vet (like everyone), because I didn't know that being a scientist was an option! Then, as I became more aware of the climate and environmental crisis, I knew I wanted to be part of the change. In the first year of my Bachelor's, one of my lecturers introduced me to ecotoxicology. At the time, he was working on the monitoring and conservation of endangered raptors, and his work fascinated me just as much as those documentaries had when I was a kid. That's when I decided this would be my career. Since then, each project has taken me to a smaller scale, from whole animals to whole physiological mechanisms and, finally, to mitochondria! I get to create my own pathway.

**M.O.:** As a teenager, I spent a lot of time volunteering at my local wildlife rehabilitation centre. Day after day, animals came in with problems that traced back to people: a goose tangled in fishing line, a songbird that had struck a window. I wanted to understand how human activities affect wildlife, not just treat the consequences. That curiosity changed how I saw the animals in our care and eventually led me into research. At first I was interested in every kind of human impact, but during my Bachelor's honours project I got to dig into the mechanisms behind contaminant effects and realised that was what I loved. Now I get to follow those effects all the way down into the cells of wild birds.

**Figure:**
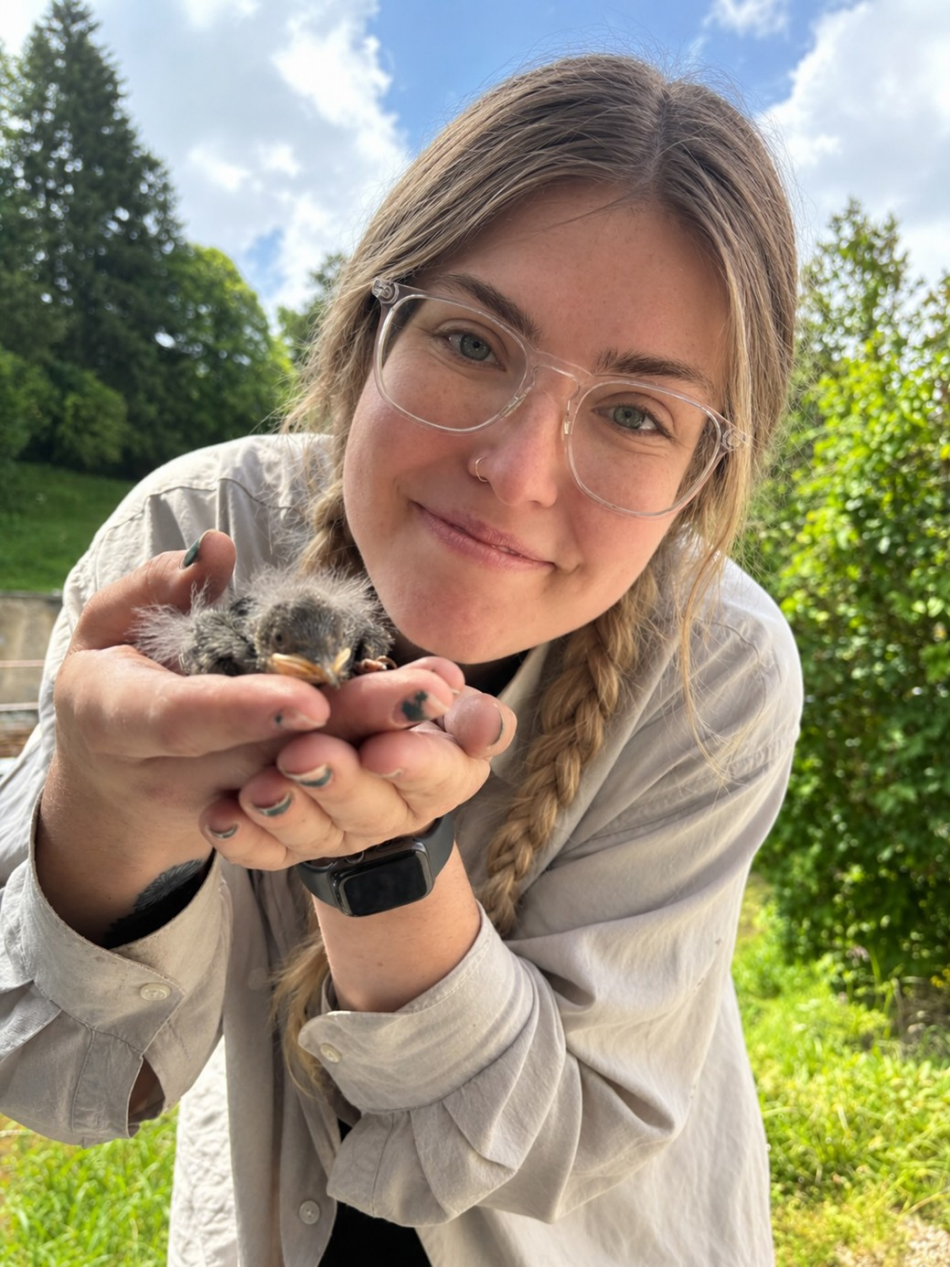
Molly Ohse

**How would you explain the main finding of your paper?**

Inside our cells are tiny power plants called mitochondria that turn food and oxygen into energy. Birds’ red blood cells, unlike ours, contain mitochondria, so a small blood sample can show how well an individual's energy production is working. By tracking how much oxygen the cells consume, we can estimate how much energy their mitochondria can produce, how much is lost as heat instead of being turned into usable energy, and how efficient they are overall. The most precise and widely used tool for measuring this can only handle a couple of samples at a time, and each run takes about two hours, which makes large studies of wild animals time-consuming. We tested a faster, less expensive approach that uses a common lab instrument to measure many samples at once and compared it side by side with this ‘gold standard’ method using blood from quails and chickens. At normal bird body temperature, the two methods broadly agreed: both showed that quails’ mitochondria used more oxygen than chickens’, and both found that male and female quails differed in how efficiently their mitochondria produce energy. However, our new approach was less sensitive, so it had trouble picking up small changes in oxygen consumption, and it was less reliable when cells were measured above or below normal bird body temperature. It can't replace the gold standard method, but it can be a useful partner for studies that need to measure many animals quickly.

**What are the potential implications of this finding for your field of research?**

Measuring mitochondrial function has helped explain why individuals differ in growth, reproduction, and survival, but with the gold standard method, high-resolution respirometry, building large datasets is slow and labour-intensive. Only a few samples can be run at a time, and blood must be measured within hours of sampling. Our results suggest that, at normal body temperature, a plate-reader approach can recover the same biological patterns as high-resolution respirometry while processing many more samples at once. Because plate readers are already common in most labs and running costs are low, this could make mitochondrial measurements accessible to more research groups, including those working on wild populations with limited budgets. Larger sample sizes would make it more practical to estimate how heritable mitochondrial traits are, compare many species, link mitochondrial function to fitness in the wild, or test how environmental contaminants affect mitochondria across a range of doses. The protocol can also be adapted and optimised for each study system; for example, adding more cells per well could improve the detection of small changes in oxygen use, and further refinements may make the approach more reliable across a wider range of temperatures. For now, the approach isn't a replacement for high-resolution respirometry, especially when small differences or extreme conditions are of interest, so the right method will depend on the question being asked.

**Figure:**
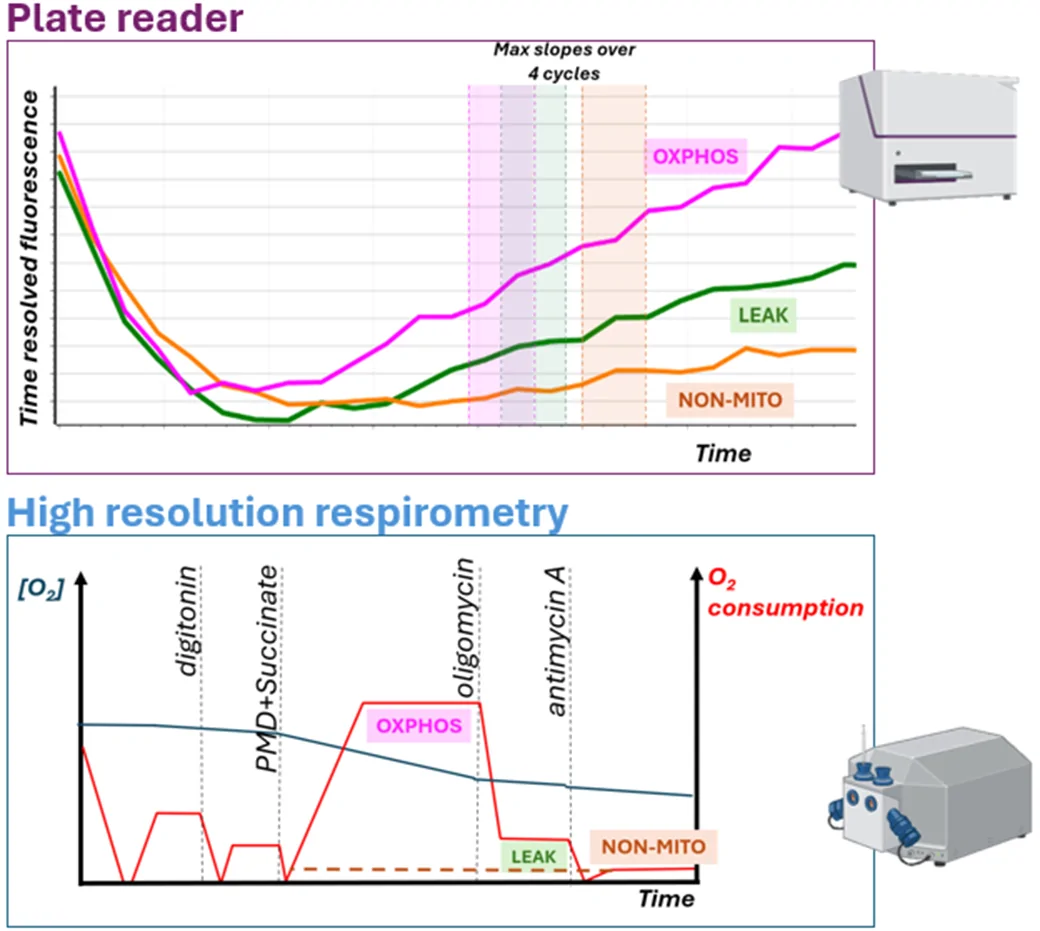
**Schematic comparison of mitochondrial respiration measurements in permeabilised avian blood cells using a fluorescence-based plate-reader assay (top) and high-resolution respirometry (bottom).** In the plate reader, OXPHOS, LEAK and non-mitochondrial respiration are measured simultaneously in separate wells from the maximum slope of MitoXpress™ Xtra fluorescence, whereas in high-resolution respirometry they are measured sequentially in a single chamber following stepwise titration of substrates and inhibitors.

**Which part of this research project was the most rewarding?**

For both of us, the most rewarding part was sharing a method that other researchers can pick up and start using straight away. Mitochondrial measurements are often out of reach for groups with limited funding, especially those working on wild animals, so we wanted to make the approach as accessible as possible. Alongside the paper, we published the full step-by-step protocol, the spreadsheets for calculating respiration rates, our R scripts, and the data. Knowing that another group could download everything and measure their own samples made the project feel worthwhile.

**What do you enjoy most about being an early-career researcher?**

**C.H.:** As an early-career researcher, I think the coolest thing is that you get to ask your own questions, be creative in how you tackle them, and eventually answer them, which usually leads to even more questions! Throughout that process, you get to collaborate with people from your own field and from completely different ones. My PhD wouldn't have been the same without the people I met along the way, including my friend and PhD teammate Molly! I also really enjoy how multidisciplinary research can be, and how it challenges me every day.

**M.O.:** What I enjoy most is how varied the work is. One month I'm standing in freezing rivers in the French Alps, catching dippers; the next I'm in a busy lab processing blood and feather samples; then I'm back at my desk working through data and writing. Each part needs different skills, and no two months ever look the same.

**What piece of advice would you give to the next generation of researchers?**

**C.H.:** Research, and the world around it, can be scary and stressful, and at times you might feel a bit lonely. When that happens, remember what brought you here in the first place. Don't forget to have fun, stay curious and open-minded!

**M.O.:** Try a bit of everything (fieldwork, lab work, statistics, writing), even the parts that feel outside your comfort zone or that you think you won't like. Take the time to learn the less glamorous skills properly, like data management, coding, and keeping good notes. They save you so much time later and make your work more reliable and easier for others to reuse. Having a broad skill set makes you more adaptable and opens doors you might not expect, both inside and outside academia.

**What's next for you?**

We are both now in the final year of our PhDs, so the coming months will be filled with analysing the lab and field data we've collected over the past three years and writing our thesis chapters before preparing for our defences.

## References

[BIO062968C1] Hadjadji, C., Ohse, M., Stier, A. and Bize, P. (2026). High-throughput mitochondrial aerobic metabolism measurement: assessing a plate-reader approach using avian blood cells. *Biol. Open.* 15, bio062712. 10.1242/bio.062712 PMC13730128 42803112

